# In Vitro Antifungal Susceptibility of* Candida* Species Isolated from Iranian Patients with Denture Stomatitis

**DOI:** 10.1155/2018/3086586

**Published:** 2018-05-16

**Authors:** Saeid Mahdavi Omran, Maryam Rezaei Dastjerdi, Maryam Zuashkiani, Vahid Moqarabzadeh, Mojtaba Taghizadeh-Armaki

**Affiliations:** ^1^Infectious Diseases and Tropical Medicine Research Center, Department of Medical Parasitology and Mycology, Babol University of Medical Sciences, Babol, Iran; ^2^Department of Prosthodontics, Faculty of Dentistry, Babol University of Medical Sciences, Babol, Iran; ^3^Department of Biostatistics, Faculty of Health, Student Research Committee, Mazandaran University of Medical Sciences, Sari, Iran; ^4^Department of Medical Mycology and Parasitology, School of Medicine, Babol University of Medical Sciences, Babol, Iran

## Abstract

**Background:**

* Candida*-associated denture stomatitis (CADS) is a common fungal infection in people who wear dentures. The main objective of this study was to make molecular identification of causative agents of CADS and in vitro antifungal susceptibility testing (AFST) in the Iranian patients with denture stomatitis.

**Methods:**

A total of 134* Candida* spp. were obtained from patients with denture stomatitis. The* Candida* spp. were identified using a polymerase chain reaction-restriction fragment length polymorphism (PCR-RFLP) involving the universal internal transcribed spacer (ITS1 and ITS4) primers, which were subjected to digestion with MspI and BlnI restriction enzymes. The in vitro antifungal susceptibility of* Candida* spp. to fluconazole (FLC), terbinafine (TRB), itraconazole (ITC), voriconazole (VRC), posaconazole (POS), ketoconazole (KET), amphotericin B (AMB), and caspofungin (CAS) was evaluated using the Clinical and Laboratory Standards Institute M27-A3 and M27-S4 guidelines.

**Results:**

Overall,* C. albicans* was the most commonly isolated species (*n* = 84; 62.6%), followed by* C. glabrata* (*n* = 23; 17.2%),* C. tropicalis* (*n* = 16; 12%), and* C. parapsilosis* (*n* = 11; 8.2%). Posaconazole had the lowest geometric mean minimum inhibitory concentration (MIC) (0.03 *μ*g/ml), followed by AMB (0.05 *μ*g/ml), ITC (0.08 *μ*g/ml), VRC (0.11 *μ*g/ml), CAS (0.12 *μ*g/ml), KET (0.15 *μ*g/ml), and FLC (0.26 *μ*g/ml).

**Discussion:**

Our study showed that* C. albicans *was most prevalent in Iranian patients with CADS and was susceptible to both azoles and amphotericin B. In addition, POS could be an appropriate alternative to the current antifungal agents used for the treatment of CADS, as well as in the treatment of recurrent candidiasis.

## 1. Introduction

*Candida*-associated denture stomatitis (CADS) is a chronic atrophic complication of the oral cavity that mainly affects people who wear removable dentures [1]. Several evidence-based studies have shown that* Candida albicans* is the main etiological agent of denture stomatitis (DS), followed by* C. tropicalis*,* C. parapsilosis*, and* C. glabrata *[2–4]. The early diagnosis of pathogenic fungal agents and the determination of their susceptibility to antifungal drugs are critical to the treatment of the infection and to establish preventive healthcare-associated strategies [5, 6].

In recent years, non-*albicans Candida *infections and antifungal resistant isolates have increased; thus, developing a reliable diagnostic method is essential for the management of candidiasis [7, 8].

A molecular-based method, such as polymerase chain reaction-restriction fragment length polymorphism (PCR-RFLP), is a promising technique that is used in the identification of pathogenic* Candida* spp. [9].

The management of CADS depends on a wide-ranging treatment strategy [10], which includes detecting and eradicating possible significant risk factors, preventing a systemic* Candida* infection, and reducing any associated discomfort [11, 12]. The use of oral formulations of antimicrobial agents, such as amphotericin B (AMB), nystatin (NYS), and miconazole (MIC), and systemic drugs, such as fluconazole (FLC), voriconazole (VRC), posaconazole (POS), itraconazole (ITC), and ketoconazole (KET), has been shown to be effective in the treatment of CADS [13–16]. Echinocandins, such as caspofungin (CAS), are a class of antifungal drugs that appear to be highly effective against all* Candida* spp., including those that are less sensitive or are resistant to FLC and/or ITC [15]. However, previous studies have described the recurrence and clinical relapse of CADS after treatment [1, 17, 18]. Having sufficient information about the antifungal susceptibility testing (AFST) of the* Candida* spp. involved in CADS may help in the selection of alternative antifungal treatments for recurrent oral candidiasis. In the current study, we evaluated the in vitro AFST of a collection of molecularly identified* Candida* spp. isolated from Iranian patients with DS.

## 2. Materials and Methods

### 2.1. Sample Collection Process

After an examination of the oral cavity, denture samples were obtained by scraping sterile swabs across the inner surface of the denture. In a period of 3 years (2013 to 2016), a total of 134 clinical isolates were collected from 103 patients aged 53–86 years affected with DS. All samples were streaked on the Sabouraud dextrose agar (Merck, Darmstadt, Germany) and incubated at 35°C for 7 days. All suspected colonies were detected by CHROMagar* Candida* (CHROMagar, Paris, France) and PCR-RFLP methods. Each isolate was preserved in the tryptic soy broth (TSB) (Merck, Darmstadt, Germany) and then stored in the culture collection of the Department of Medical Mycology, Babol University of Medical Sciences, Iran.

### 2.2. Genomic DNA Extraction and PCR-RFLP

The total genomic DNA from the yeast was removed using the method described by Yamada et al., which involved cell disruption with glass beads followed by extraction with phenol–chloroform and precipitation with ethanol [19].

Oligonucleotide primer sequences including ITS1 (5′-TCCGTAGGTGAACCTGCGG-3′) and ITS4 (5′-TCCTCCGCTTATTGATATGC-3′) were used in this study [20]. Amplification was performed on a thermal cycler (C1000; Bio-Rad Laboratories, Inc.). The amplified products were electrophoresed on 1.5% agarose gels containing 0.5 mg/ml of ethidium bromide and then analyzed under UV light using a gel-doc system (Bio-Rad, USA). The breakdown of the amplified products involved the restriction enzymes BlnI and/or MspI (Table 1). The digests of the PCR fragments were electrophoresed on 1.5% agarose gels. In this study,* C. albicans *ATCC 10231 and* C. dubliniensis *CBS 2747 were used as quality control strains.

### 2.3. Antifungal Susceptibility Testing

The following antifungal agents were evaluated: AMB (Bristol-Myers Squib, Woerden, Netherlands), FLC, ITC, VRC, KET, and TRB (Sigma-Aldrich, St. Louis, MO, USA), POS (Schering-Plough Corp., Oss, Netherlands), and CAS (Pfizer, Capelle aan den Ijssel, Netherlands). In vitro AFST was performed according to the Clinical and Laboratory Standards Institute (CLSI) M27-A3 and M27-S4 guidelines [21, 22]. Each antifungal agent was prepared separately. The final concentration of FLC ranged from 0.063 to 64 *μ*g/ml. The final concentrations of AMB, ITC, VRC, POS, and KET ranged from 0.016 to 16 *μ*g/ml, while the final concentrations of CAS and TRB were 0.008–8 *μ*g/ml and 0.12–128 *μ*g/ml, respectively. The drugs were diluted in RPMI-1640 Medium (Sigma-Aldrich, Darmstadt, Germany) and buffered to pH 7.0 with 0.165 M N-morpholinepropanesulfonic acid (MOPS) (Sigma-Aldrich, USA) and L-glutamine without bicarbonate to yield twofold their final concentrations. The primary* Candida* spp. were cultured on potato dextrose agar (PDA; Difco, Leeuwarden, Netherlands) and incubated for 3 days at 35°C. Once mature colonies were observed, a conidial inoculum was made using a sterile saline solution. A spectrophotometer at 530 nm was used to adjust the inoculum to a range of 2.5–5 × 10^6^ CFU/ml. The drug containing 96-well plastic microplates was inoculated with this suspension and incubated at 35°C for 24–48 h. The minimum inhibitory concentrations (MICs) for FLC, VRC, CAS, ITC, and POS were determined according to the CLSI M27-A3 and M27-S4 guidelines [21, 22]. Isolates that responded to ≤1 *μ*g/ml MIC for AMB were recognized as susceptible isolates according to the CLSI M27-S3 guideline [23]. The breakpoint was not determined for TRB; however, several studies have reported resistance breakpoints ≥ 8 *μ*g/ml [24, 25]. The breakpoint values for KET were not defined by the CLSI and, thus, the resistant breakpoint of ≥4 *μ*g/ml which was determined by Mulu et al. (2013) was used [26]. Isolates from* C. krusei* (ATCC 6258) and* C. parapsilosis* (ATCC 22019) were used as quality control strains.

### 2.4. Data and Statistical Analysis

The geometric mean (GM), MIC_50_, and MIC_90_ for the antifungal agents against* Candida* spp. were calculated using EXCEL (Microsoft Office Excel 2003 SP3, Microsoft Corporation, Redmond, USA).

## 3. Results

*C. albicans* was the predominant species (*n* = 84; 62.6%), followed by* C. glabrata* (*n* = 23; 17.2%),* C. tropicalis* (*n* = 16; 12%), and* C. parapsilosis* (*n* = 11; 8.2%).  Table 2 summarizes the GM of the MICs, the MIC ranges, MIC_50_, and MIC_90_ for the antifungal drugs against all* Candida *isolates. The GM of MICs for drugs across all strains was, in increasing order, 0.03 *μ*g/ml (POS), 0.05 *μ*g/ml (AMB), 0.08 *μ*g/ml (ITC), 0.11 *μ*g/ml (VRC), 0.12 *μ*g/ml (CAS), 0.15 *μ*g/ml (KET), 0.26 *μ*g/ml (FLC), and 65.00 *μ*g/mL (TRB). All* C. albicans* isolates (100%) were found to be susceptible to AMB, VRC, POS, KET, and ITC; however, 13 isolates (15.5%) were resistant to FLC. All* C. parapsilosis* isolates (100%) were susceptible to FLC, while only 4 isolates (17.4%) of* C. glabrata* and 2 isolates (12.5%) of* C. tropicalis* were resistant to FLC. The resistance rates for VRC of* C. tropicalis, C. parapsilosis,* and* C. glabrata* were 18.7% (3/16), 8.6% (2/23), and 9.1% (1/11), respectively. The ITC MICs for 6 isolates (37.5%) of* C. tropicalis* and 4 isolates (36.4%) of* C. *parapsilosis were ≥1 *μ*g/ml. The resistance rates for AMB in* C. tropicalis* and* C. parapsilosis *were 12.5% (2/16) and 45.5% (5/11), respectively. Out of 134 isolates, 1 isolate of* C. tropicalis* (≥4 *μ*g/ml) was resistant to KET. The resistance rates for CAS in* C. glabrata*,* C. tropicalis*, and* C. albicans* were 56.5% (13/23), 9.1% (1/11), and 2.3% (2/84), respectively. Overall, all* Candida* spp. had the highest in vitro antifungal susceptibility to ITC, POS, and CAS. However,* Candida* spp. showed a lack of susceptibility to TRB.

## 4. Discussion

Dentures in the oral cavity are considered to be a reservoir of* Candida* spp. and, thus, are a predisposing factor for DS in patients, as well as a potential origin of reinfection [27]. CADS is an infection initiated by the oral colonization of* Candida *spp.; the most frequently identified species is* C. albicans*, although* C. glabrata*,* C. guilliermondii*,* C. parapsilosis*,* C. krusei*, and* C. tropicalis* are less commonly seen [28, 29]. In agreement with other studies, our research found that* C. albicans*,* C. parapsilosis*,* C. tropicalis*, and* C. glabrata* caused CADS [30–32]. The recommended drug of choice to treat CADS in patients without an underlying disease commonly includes a NYS suspension or a clotrimazole tablet. However, a topical application of an azole, such as FLC or ITC, can also be used to prevent persistent or chronic fungal infections in the patients [33, 34].

Several studies reported the emergence of antifungal resistance to azoles, which has been associated with multiple episodes of recurrence [16, 35–37]. In the current study, 15.5% of* C. albicans* (13/84) was observed to be resistant to FLC. In contrast with our data, Abaci and Haliki-Uztan (2011) reported that 59.4% of* C. albicans* were resistant to FLC [24].

AMB, also used in the management of CADS, proved effective against* Candida* spp. [1]. Besides, the findings obtained in the present study were in agreement with the results by Wingeter et al. (2007) [38] regarding the susceptibility of oral* Candida* strains to AMB.

AMB-resistant non-*albicans* isolates were reported from several previous studies [24, 39]. We also found that 12.5% (2/16) of* C. tropicali*s and 45.5% (5/11) of* C. parapsilosis* isolates showed resistance patterns to AMB. The good in vitro activities of POS and VRC have been previously reported against* Candida* spp. obtained from oral candidiasis patients [40–43].

As shown in Table 2, POS was the most effective drug in vitro with GM MICs of 0.01 *μ*g/ml, 0.19 *μ*g/ml, 0.05 *μ*g/ml, and 0.05 *μ*g/ml for* C. albicans*,* C. glabrata*,* C. tropicalis*, and* C. parapsilosis, *respectively. Marcos-Arias et al. (2012) previously showed that the GM MICs for POS were 0.036 *μ*g/ml for* C. parapsilosis*, 0.062 *μ*g/ml for* C. albicans*, 0.085 *μ*g/ml for* C. tropicalis*, and 0.498 *μ*g/ml for* C. glabrata* [16]. Several other studies also demonstrated that POS and VRC were strong antifungal agents against* Candida* spp. [40–44].

In our study, all non-*albicans Candida* isolates were susceptible to POS, although only 88% these isolates were susceptible to VRC. In line with the Marcos-Arias et al. (2012), VRC was effective against 95.5% of strains [16]. In addition, the GM MICs for ITC were 0.04 *μ*g/ml for* C. albicans*, 0.26 *μ*g/ml for* C. glabrata*, 0.27 *μ*g/ml for* C. tropicalis*, and 0.46 *μ*g/ml for* C. parapsilosis*. Other studies have shown that ITC is useful for treating patients with DS [27, 45, 46].

Dorocka-Bobkowska and Konopka (2007) reported that AMB, FLC, and ITC were effective against 100%, 88.7%, and 87.3% of* C. albicans* and 79.6%, 71.4%, and 79.6% of other* Candida* strains, respectively [10]. In the present study, AMB, FLC, and ITC were effective against 100%, 84.5%, and 100% of* C. albicans* and 86%, 88%, and 80% of non-*albicans Candida *isolates, respectively. Caspofungin is known as an echinocandin fungicidal antifungal agent against most* Candida* spp. [15].

Some data are available on the AFST of* Candida* spp. isolated from denture-associated stomatitis (DAS) to echinocandins [15, 47]. In the present study, only 2 isolates (2.3%) of the 84 isolates of* C. albicans* were resistant to CAS. We also found that 14 isolates (28%) of the non-*albicans Candida* strains were resistant to CAS.

In the present study, TRB was not found to be effective against* Candida* spp. Ryder et al. (1998) also reported that TRB was not an active drug against *C. glabrata and C. tropicalis* [25].

Our results revealed that the tested antifungal showed good activity for most isolates; however, variability observed among some isolates and resistance to drugs highlight the need for AFST as a monitor to management of therapeutic procedure.

## 5. Conclusion

In conclusion, all* Candida* spp. isolated from patients wearing dentures were susceptible to POS and AMB. As an antifungal, POS could be a suitable alternative to the present antifungal agents used for the management of CADS and could be also used in the treatment of recurrent candidiasis.

## Figures and Tables

**Table 1:** The cutting size PCR-products of ITS region for different *Candida *spp.subjected to digestion with *MspI* and *BlnI* restriction enzymes.

*Candida* species	Size of ITS1-ITS4, bp	Size (s) of restriction product (s), bp	
		MspI	BlnI
*C. albicans*	535	297, 238	535
*C. glabrata*	871	557, 314	
*C. tropicalis*	524	340, 184	
*C. parapsilosis*	520	520	
*C. dubliniensis*	535	297, 238	200–335

**Table 2:** In vitro antifungal susceptibility of eight antifungal agents against 134 *Candida* spp. isolated from *Candida*-associated denture stomatitis.

*Candida* species/number of strains/antifungal drugs	MIC *µ*g/mL			
	MIC range	MIC50	MIC90	GM
*All Candida species (134)*				
FLC	0.016–16	0.125	8	0.26
ITC	0.016–16	0.064	0.5	0.08
VRC	0.016–4	0.125	0.5	0.11
AMB	0.016–2	0.064	0.25	0.05
CAS	0.008–2	0.125	0.5	0.12
TRB	2–≥128	128	>128	65.00
POS	0.032–0.5	0.016	0.125	0.03
KET	0.016–4	0.125	1	0.15
*C. albicans *(84)				
FLC	0.016–16	0.064	2	0.09
ITC	0.016–0.5	0.032	0.5	0.04
VRC	0.032–0.25	0.064	0.25	0.08
AMB	0.008–0.25	0.032	0.25	0.03
CAS	0.008–1	0.064	0.5	0.08
TRB	2–≥128	128	128	96.68
POS	0.032–0.5	0.016	0.032	0.01
KET	0.016–2	0.125	1	0.09
*C. glabrata* (23)				
FLC	0.25–≥16	4	64	5.24
ITC	0.125–0.5	0.25	0.5	0.26
VRC	0.125–2	0.25	0.5	0.24
AMB	0.032–0.5	0.032	0.5	0.07
CAS	0.008–2	0.5	1	0.51
TRB	8–>128	16	64	19.77
POS	0.125–0.5	0.125	0.5	0.19
KET	0.064–1	0.125	0.5	0.14
*C. tropicalis* (16)				
FLC	0.125–≥16	0.125	4	0.42
ITC	0.016–16	0.016	4	0.27
VRC	0.016–4	0.032	2	0.08
AMB	0.016–2	0.125	1	0.17
CAS	0.008–1	0.032	0.125	0.05
TRB	4–≥128	64	128	53.81
POS	0.016–0.25	0.032	0.25	0.05
KET	0.032–4	0.25	2	0.23
*C. parapsilosis* (11)				
FLC	0.25–4	0.25	2	0.46
ITC	0.125–8	0.25	4	0.46
VRC	0.25–2	0.25	1	0.41
AMB	0.016–2	0.25	1	0.19
CAS	0.008–2	0.25	1	0.41
TRB	4–≥128	128	128	49.74
POS	0.032–0.125	0.032	0.125	0.05
KET	0.125–2	0.125	1	0.25

FLC, fluconazole; ITC, itraconazole; VRC, voriconazole; AMB, amphotericin B; CAS, caspofungin; TRB, terbinafine; POS, posaconazole; KET, ketoconazole; GM, geometric mean; MIC, minimum inhibition concentration; MIC50 and MIC90, concentration at which 50% and 90% of the strains were inhibited, respectively.

## Data Availability

The data used to support the findings of this study are available from the corresponding author upon request.
